# Relevance of Carcinogen-Induced Preclinical Cancer Models

**DOI:** 10.3390/jox14010006

**Published:** 2024-01-05

**Authors:** Raj N. Sewduth, Konstantina Georgelou

**Affiliations:** 1 VIB KU Leuven Center for Cancer Biology, 49 Herestraat, 3000 Leuven, Belgium; 2 Institute of Molecular Biology and Biotechnology (IMBB), Foundation for Research and Technology—Hellas (FORTH), N. Plastira 100, Vasilika Vouton, GR-70013 Heraklion, Greece

**Keywords:** carcinogenic agents, xenobiotics, cancer models, clinical relevance

## Abstract

Chemical agents can cause cancer in animals by damaging their DNA, mutating their genes, and modifying their epigenetic signatures. Carcinogen-induced preclinical cancer models are useful for understanding carcinogen-induced human cancers, as they can reproduce the diversity and complexity of tumor types, as well as the interactions with the host environment. However, these models also have some drawbacks that limit their applicability and validity. For instance, some chemicals may be more effective or toxic in animals than in humans, and the tumors may differ in their genetics and phenotypes. Some chemicals may also affect normal cells and tissues, such as by causing oxidative stress, inflammation, and cell death, which may alter the tumor behavior and response to therapy. Furthermore, some chemicals may have variable effects depending on the exposure conditions, such as dose, route, and duration, as well as the animal characteristics, such as genetics and hormones. Therefore, these models should be carefully chosen, validated, and standardized, and the results should be cautiously interpreted and compared with other models. This review covers the main features of chemically induced cancer models, such as genetic and epigenetic changes, tumor environment, angiogenesis, invasion and metastasis, and immune response. We also address the pros and cons of these models and the current and future challenges for their improvement. This review offers a comprehensive overview of the state of the art of carcinogen-induced cancer models and provides new perspectives for cancer research.

## 1. Introduction

Preclinical cancer models are essential tools for studying the mechanisms of carcinogenesis and testing new therapeutic strategies [1,2,3]. However, not all models can faithfully recapitulate the complexity and heterogeneity of human cancers induced by carcinogenic agents [4,5]. Some of the hallmarks of carcinogen-induced preclinical cancer models are that they are derived from animals that have been exposed to known or suspected carcinogens, such as chemical agents, radiation, viruses, or transgenic oncogenes. These exposures can induce different types and frequencies of mutations, depending on the nature, dose, and duration of the carcinogen, as well as the susceptibility and genetic background of the animal [6,7,8,9,10]. Carcinogen-induced preclinical cancer models also exhibit a high degree of genetic and epigenetic instability, resulting in multiple mutations and alterations in key pathways involved in cancer development and progression. These include oncogenes, tumor suppressor genes, DNA repair genes, cell cycle regulators, apoptosis mediators, and epigenetic modifiers. The accumulation of these changes can confer selective advantages to the tumor cells, such as increased proliferation, survival, invasion, and metastasis, in a process similar to the one observed in patients [11,12,13].

The carcinogen-induced preclinical cancer models are influenced by the microenvironment and host factors, such as stromal cells, immune cells, hormones, and cytokines, that modulate tumor growth and response to treatment [14,15,16]. The interactions between the tumor cells and their surroundings can create a dynamic and reciprocal feedback loop that can promote or inhibit tumorigenesis. The microenvironment and host factors can also affect the pharmacokinetics and pharmacodynamics of anticancer agents, as well as their toxicity and side effects. 

These models can also be used to study the effects of different doses and schedules of carcinogens, as well as the interactions between multiple carcinogens and their synergistic or antagonistic effects. This can help to elucidate the dose–response relationship and the threshold level of carcinogen exposure that can induce tumorigenesis. It can also help to identify the most potent and relevant carcinogens for human cancers, as well as the potential preventive or protective measures that can reduce their risk. They can be used to evaluate the efficacy and toxicity of novel anticancer agents, alone or in combination, as well as the mechanisms of resistance and biomarkers of response. This can help to optimize the drug development process and to select the most promising candidates for clinical trials in carcinogen-induced human cancers, such as smoking induced lung cancer, liver cancer, or colorectal cancer. It could also elucidate the molecular mechanisms of action and resistance of anticancer agents as well as identify predictive and prognostic biomarkers that can guide personalized medicine.

These models also display a range of phenotypic and histopathological features that resemble human cancers, such as invasion, metastasis, angiogenesis, inflammation, and immune evasion [17]. These features can vary depending on the organ and tissue of origin, the stage and grade of the tumor, and the molecular subtype and profile of the tumor. Some models can also mimic the clinical course and outcome of human cancers, such as response to treatment, recurrence, and survival. 

However, clear limitations of carcinogen-induced preclinical models should be known and described in an extensive manner, as such information could make it possible to choose the most appropriate model for drug testing and enable a proper translation of preclinical data to patients presenting cancers caused by xenobiotics. For example, some chemicals may be more potent and effective in inducing cancer in animals than in humans, and the resulting tumors may have different genetic and phenotypic characteristics. Some chemicals may also have non-specific effects on normal tissues and cells, such as oxidative stress, inflammation, and apoptosis, which may indirectly influence the tumor growth, progression, and response to therapy. Moreover, some chemicals may have different effects depending on the dose, route, and duration of exposure, as well as the genetic background and hormonal status of the animals. Therefore, carcinogen-induced preclinical cancer models should be carefully selected, validated, and standardized, and the results should be interpreted with caution and compared with other models [18]. In this review, we summarize the hallmarks of preclinical cancer models driven by carcinogenic agents such as genetic and epigenetic alterations, tumor microenvironment, angiogenesis, invasion and metastasis, and immune response. We also discuss the advantages and limitations of these models, as well as the current challenges and future directions for their improvement. This review provides a comprehensive overview of the state of the art of carcinogenic cancer models and inspire new insights into cancer research.

## 2. Examples of Chemical Carcinogens in Mouse Cancer Models

Some examples of chemical carcinogens that are commonly used to induce mouse cancer models are listed in the table below (Table 1). Mice are often used as a model for studying the effects of environmental carcinogens because they share many genetic and physiological similarities with humans. However, it is important to note that the results of animal studies may not always be directly applicable to humans, and further research is needed to fully understand the health effects of exposure to environmental carcinogens. 

The compounds listed in Table 1 can be detected in the environment and can directly affect human health. However, the concentrations of these compounds in the environment may vary depending on the location and the source of contamination. For example, urethane is a widely used solvent and industrial chemical that can cause cancer in humans. It has been detected in the air, water, and soil in some areas. Diethylnitrosamine (DEN) is found in tobacco smoke. Dimethylbenz(a)anthracene (DMBA) is a potent carcinogen for mice and humans and is used to specifically quantify phlorotannins. Dextran sulfate sodium (DSS) is a common environmental pollutant and dietary component that can cause inflammation and cancer in humans. Azoxymethane (AOM) is a widely used carcinogen to study chemically induced colorectal carcinogenesis and is an agent for studying fulminant hepatic failure. Cerulein (or ceruletide or caerulein) is present in the skin of the Australian green tree frog and is used in paralytic ileus and as a diagnostic aid in pancreatic malfunction.

### 2.1. AOM

**Figure 1:**
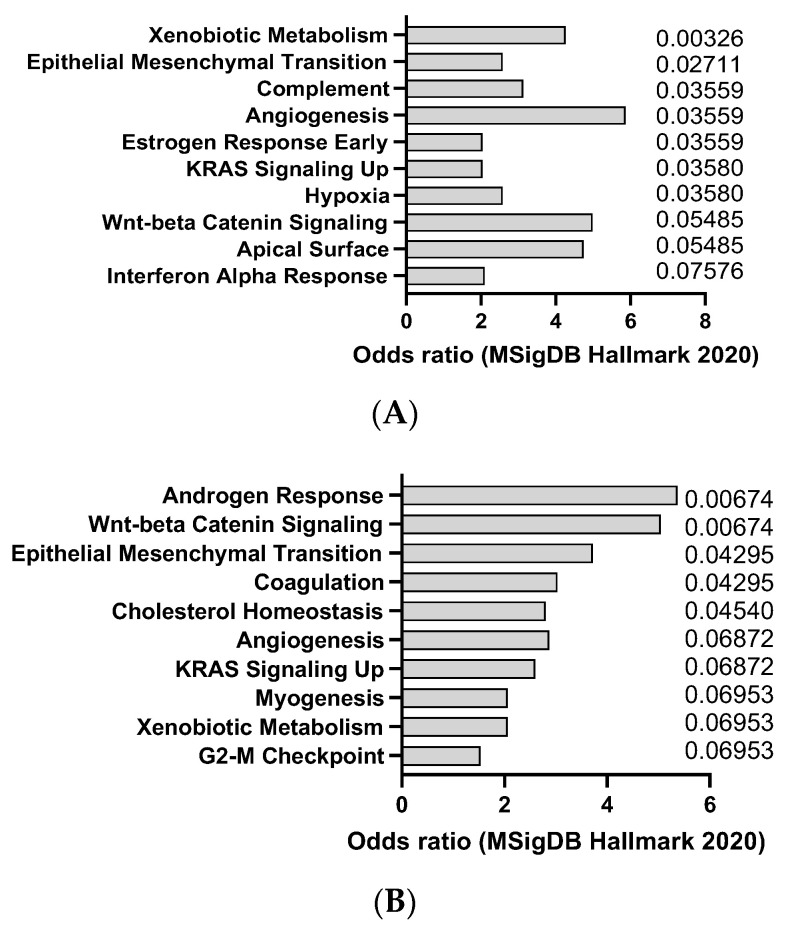
mSigDB Hallmark 2020 pathway analysis of transcriptomics changes detected in AOM-induced dysplasia (8 weeks after treatment) (**A**) and colorectal cancer in mice (20 weeks after treatment) (**B**), sorted by adjusted *p*-value (data adapted from GEO dataset GSE31106).

AOM is a colon-specific carcinogen that can induce colorectal carcinoma in mice, especially in combination with DSS, an inflammatory agent that causes colitis. AOM can induce DNA methylation and mutations in oncogenes and tumor suppressor genes, such as Apc, Kras, and Tp53, that are frequently mutated in human colorectal cancer (CC). This was confirmed by transcriptomics analysis of dysplasia (8 weeks after AOM) and CC (20 weeks after AOM), showing a trend towards activation of K-ras signaling (Figure 1A,B). The model also appears to recapitulate, at least at early stage, interferon alpha response, which is usually observed in CC patients. AOM is usually administered intraperitoneally at doses ranging from 5 to 15 mg/kg and can induce tumors within 3 to 6 months [24]. AOM-induced colon tumors are highly heterogeneous and can be classified into different histological subtypes, such as tubular, tubulovillous, and villous. AOM-induced colon tumors can also metastasize to other organs, such as the liver, lung, and lymph nodes [25]. The AOM model is a widely used model of experimental colon carcinogenesis in rodents.

However, this model also has some limitations, such as genetic-background-dependent effects [26]. For example, different mouse strains show different susceptibility and response to AOM treatment, which may affect the reproducibility and comparability of the results. AOM also does not induce microsatellite instability, which is a hallmark of human CC. Furthermore, high dose and long duration of AOM are required to induce CC. AOM treatment requires high doses (10–15 mg/kg) and long duration (20–30 weeks) to induce tumors in rodents, which may not reflect the natural course of human CC. Finally, AOM-induced tumorigenesis can be modulated by various dietary and environmental factors, such as fiber, fat, antioxidants, inflammation, and gut microbiota. These factors may introduce variability and confounding effects in the results [27]. Novel models of colitis were recently proposed, showing an interest in models that better reproduce human CC [28].

### 2.2. Cerulein

**Figure 2:**
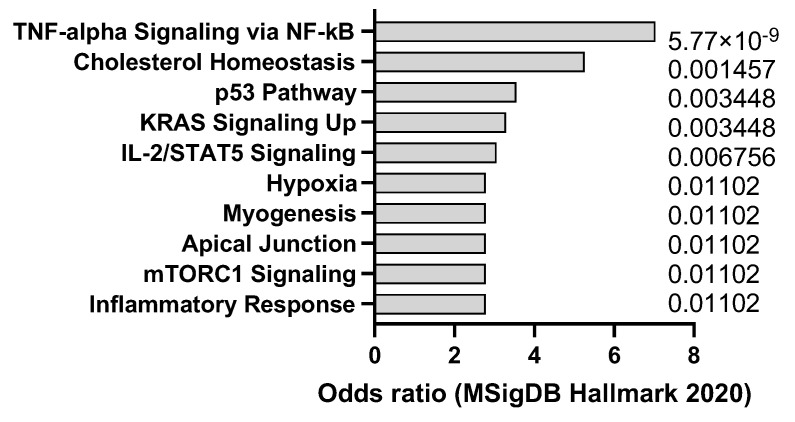
mSigDB Hallmark 2020 pathway analysis of transcriptomics changes detected in cerulein-induced pancreatitis in mice, sorted by adjusted *p*-value (data adapted from GEO dataset GSE3644).

Cerulein is a peptide that can induce acute or chronic pancreatitis in mice by stimulating the secretion of pancreatic enzymes and causing inflammation and fibrosis. Pancreatitis is a risk factor for pancreatic ductal adenocarcinoma (PDAC), a highly lethal and aggressive type of cancer that originates from the cells lining the pancreatic ducts [29]. Mouse models of PDAC can be generated by using genetic engineering or chemical carcinogens to activate oncogenic mutations in pancreatic cells, such as KRAS, TP53, CDKN2A, and SMAD4 [30]. Transcriptomics analysis confirms that cerulein in mice drives p53 and KRAS signaling signatures but also promotes a tumor necrosis factor alpha (TNF-a) response (Figure 2) that is in line with the signatures typically observed in human pancreatitis. Cerulein can be used to enhance the tumorigenesis and progression of PDAC in mouse models by creating a pro-inflammatory and pro-fibrotic microenvironment that promotes cell proliferation, invasion, angiogenesis, and immune evasion. Mouse models of PDAC with cerulein-induced pancreatitis can be used to study the molecular mechanisms of cancer development and evaluate the effectiveness of chemoprevention and immunotherapy strategies that target the inflammatory and fibrotic pathways.

However, this model presents several limitations [31]. One of the limitations of using cerulein to induce pancreatitis in mouse models of PDAC is that it may not fully mimic the natural history and complexity of human PDAC, which can arise from different types of precursor lesions and genetic alterations. Another limitation is that cerulein can cause systemic toxicity and mortality in mice, especially at high doses and long-term administration. Therefore, the optimal dose and duration of cerulein treatment need to be carefully determined to balance the efficacy and safety of the model. A third limitation is that cerulein-induced pancreatitis may not be suitable for studying the early stages of PDAC initiation and development, as it mainly accelerates the growth and metastasis of existing tumors. Thus, cerulein should be combined with other methods to generate more realistic and diverse mouse models of PDAC that better recapitulate human disease [32].

### 2.3. DEN

**Figure 3:**
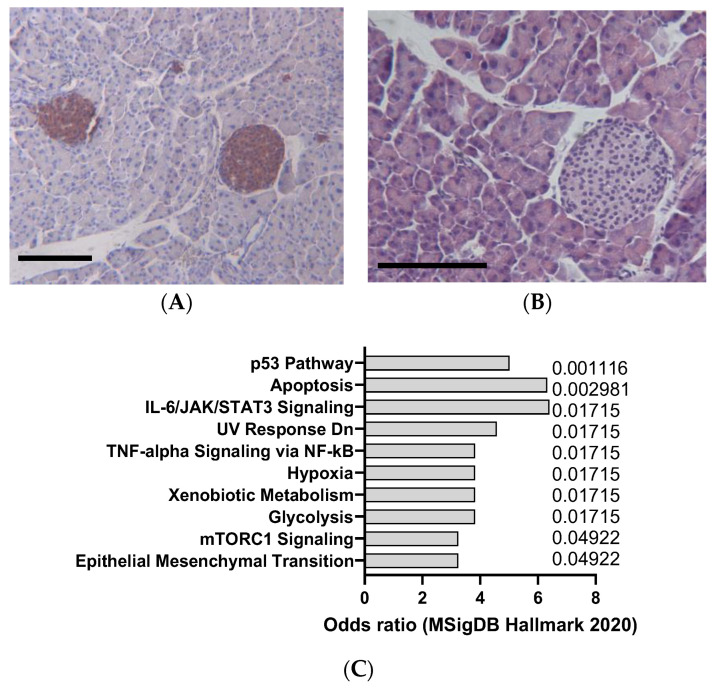
Example of liver cancer in DEN-induced animals (25 mg/kg IP), detected according to cytokeratin 19 staining (**A**) and hemalum–eosin staining (**B**). Scale bar: 100 µm. (**C**) mSigDB Hallmark 2020 pathway analysis of transcriptomics changes detected in carcinogen-induced liver cancer in mice, sorted by adjusted *p*-value (data adapted from GEO dataset GSE51358).

**Figure 4:**
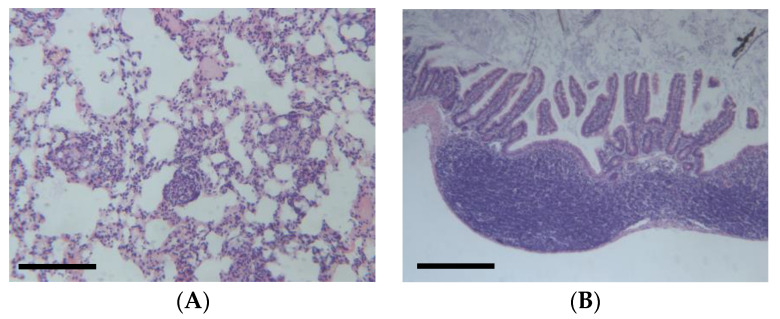
(**A**) Example of lung metastasis observed in DEN-induced animals (25 mg/kg IP), (**B**) Example of colitis in DEN-induced animals, according to hemalum–eosin staining. Scale bar: 100 µm (unpublished data).

DEN is a potent hepatocarcinogen that can induce hepatocellular carcinoma (HCC) in mice (Figure 3A,B), especially in susceptible strains such as C3H/He and C57BL/6. DEN can cause DNA damage and oxidative stress, leading to mutations and alterations in oncogenes and tumor suppressor genes, such as β-catenin, Met, and p53, that are commonly involved in human liver cancer [33]. DEN is usually administered intraperitoneally at doses ranging from 1 to 25 mg/kg and can induce tumors within 6 to 12 months, as indicated by Figure 3A,B. DEN-induced liver tumors are highly heterogeneous and can be classified into different histological subtypes, such as trabecular, pseudoglandular, and scirrhous. DEN-induced liver tumors can also metastasize to other organs, such as the lung, spleen, and lymph nodes, as demonstrated in Figure 4A. DEN is one of the most widely used chemical carcinogens to induce mouse models of HCC, as it can partially mimic the molecular and pathological features of human HCC [34]. DEN can induce HCC via two mechanisms: through direct DNA damage or by facilitating tumor formation after initiation through a hepatotoxic compound that promotes clonal expansion. DEN can also activate various signaling pathways, such as the IL-6/STAT3, Wnt/β-catenin, and Ras/MAPK pathways, that are involved in cell proliferation, survival, and inflammation. Our analysis of transcriptomics data confirms that DEN in mice appears to clearly induce interleukin 6 (IL-6)/JAK/STAT3 (Figure 3C), which is in line with the signatures typically observed in human HCC.

DEN-induced HCC can be influenced by several factors, such as the genetic background, sex, age, and diet of the mice [35]. For example, a high-fat diet can promote macrophage-mediated hepatic inflammation and aggravate DEN-induced hepatocarcinogenesis in mice. Therefore, DEN-induced mouse models can provide valuable insights into the mechanisms and therapeutic strategies of HCC. However, this model can also cause colitis (Figure 4B) and also presents several limitations [36]. For example, the dose of DEN used in the model varies from 1.25 to 100 g per kg body weight of mouse via intraperitoneal injection. Additionally, the molecular mechanisms of the differences between chemically and virus-induced HCC models remain elusive. It is worth noting that the clinical relevance of the DEN mouse model has been questioned. A study published in the *International Journal of Molecular Sciences* states that the DEN model does not accurately reflect the human disease and that the results obtained from this model may not be clinically relevant. The study suggests that researchers should use alternative models or adapt current models to better mimic human disease [37]. 

### 2.4. DMBA

DMBA is a chemical compound that belongs to the class of polycyclic aromatic hydrocarbons (PAHs), which are known to be environmental carcinogens. DMBA can induce breast cancer in mice by causing DNA damage and mutations in various tissues, especially in the mammary gland [38]. DMBA requires metabolic activation by cytochrome P450 enzymes to generate a DNA-reactive metabolite that forms DNA adducts and induces mutations and chromosomal aberrations. DMBA can also activate oncogenic signaling pathways, such as the aryl hydrocarbon receptor (AhR), the Wnt pathway, the NF-κB pathway, and the prolyl isomerase Pin-1, which can promote cell proliferation, survival, and invasion [39]. DMBA can induce different types of breast or skin tumors in mice, depending on the dose, route of administration, genetic background, and hormonal status of the animals [40]. DMBA-induced mouse mammary tumors can exhibit elevated expression of c-myc, cyclin D1, and hyperphosphorylated retinoblastoma (Rb) protein, which are involved in cell cycle regulation. DMBA can also affect the normal tissues and cells surrounding the tumor, such as the immune system, the stroma, and the vasculature, which can influence the tumor growth, progression, and response to therapy [41].

**Figure 5:**
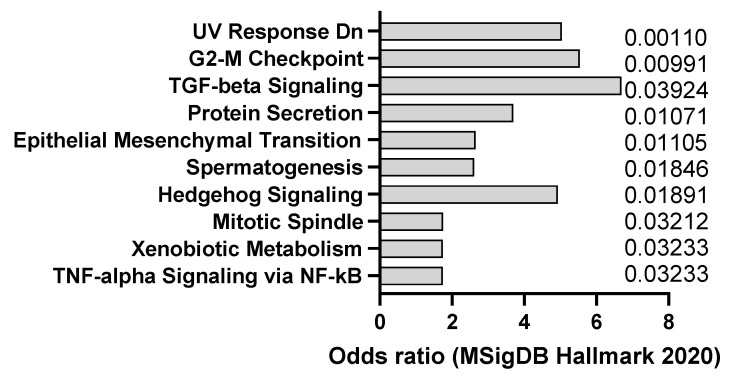
mSigDB Hallmark 2020 pathway analysis of transcriptomics changes detected in DMBA-induced breast cancer in mice, sorted by adjusted *p*-value (data adapted from GEO dataset GSE16232).

However, this model also presents several limitations, such as major species differences between mice and humans. DMBA is more potent and effective in inducing breast or skin cancer in mice than in humans, and the tumors may have different molecular and histological features. Furthermore, DMBA-induced breast cancer can present a strong heterogeneity, as DMBA can induce different types of breast tumors in mice, depending on the dose, route of administration, genetic background, and hormonal status of the animals [42]. This may affect the reproducibility and comparability of the results. DMBA has an effect on the tumor microenvironment, as DMBA can affect the normal tissues and cells surrounding the tumor, such as the immune system by blocking T-cell function, the stroma, and the vasculature. This may influence the tumor growth, progression, and response to therapy. Our analysis of transcriptomics data indicates that DMBA in breast tissue appears to induce epithelial–mesenchymal transition, associated with TGF-b activation and TNF-a signaling (Figure 5), which is in line with the signatures typically observed in human breast cancer. DMBA can also induce skin cancer in mice that shares some phenotypic overlap with the human disease [43].

### 2.5. DSS

**Figure 6:**
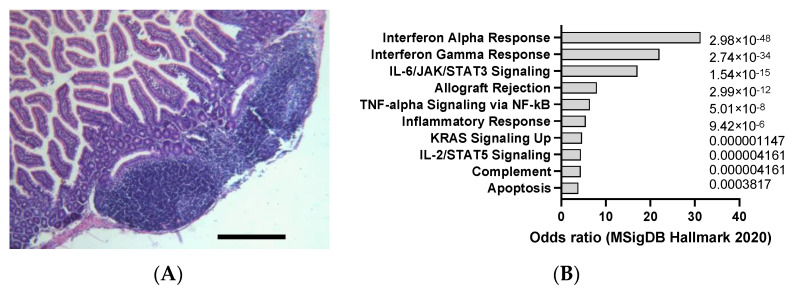
(**A**) Example of DSS-induced colitis (2% DSS solution in drinking water), according to hemalum–eosin staining. Scale bar: 100 µm (unpublished data). (**B**) mSigDB Hallmark 2020 pathway analysis of transcriptomics changes detected in DSS-induced colitis in mice (data adapted from GEO dataset nrGSE201360).

DSS mouse colitis is a widely used experimental model of a type of inflammatory bowel disease (IBD) that affects the large intestine. IBD is a chronic condition that causes inflammation and ulcers in the digestive tract, leading to symptoms such as diarrhea, abdominal pain, and weight loss, potentially causing CC when combined with genetic mutations or other chemicals [44]. Ulcerative colitis is a form of IBD that only affects the colon, also known as the large bowel. DSS stands for dextran sulfate sodium, which is a chemical compound that has a negative charge and a high molecular weight. When DSS is added to the drinking water of mice, it damages the protective layer of cells that line the colon, making it more vulnerable to bacterial invasion and immune attack [45]. This results in inflammation, ulceration, and bleeding in the colon, similar to that in human ulcerative colitis, as observed in Figure 6A. DSS mouse colitis can be induced by administering mice with DSS in their water for a certain period, usually between 5 and 7 days, depending on the dose and the strain of mice. The severity of colitis can be measured by various methods, such as measuring weight loss and colon shrinkage, examining the colon tissue for damage under a microscope, and testing the blood or colon tissue for levels of inflammatory substances that are produced by the immune system [46]. DSS mouse colitis is a useful model for studying the causes and treatments of IBD, as well as the interactions between the bacteria that live in the gut, the immune system that defends the body, and the brain that controls the behavior and mood of the mice. DSS in mice appear to induce a clear interferon alpha and gamma response as well as an interleukin 6 (IL-6) and tumor necrosis factor alpha (TNF-a) response (Figure 6B) that is in line with the signatures typically observed in human colitis.

However, this model also has some limitations, such as the dose and timing of DSS administration, that can vary depending on the mouse strain, age, sex, and diet, which can affect the reproducibility and consistency of the results [47]. The DSS model mainly induces tumors in the distal colon, whereas human CC can occur throughout the colon and rectum. The DSS model does not fully recapitulate the genetic and epigenetic alterations that occur in human CC, such as chromosomal instability, microsatellite instability, and the CpG island methylator phenotype [48]. The DSS model does not account for the role of environmental and lifestyle factors, such as obesity, smoking, alcohol, and diet, that can influence the risk and progression of human colorectal cancer. Some recent work investigated such an approach by combining DSS/AOM with a high-fat diet [49].

### 2.6. Urethane

**Figure 7:**
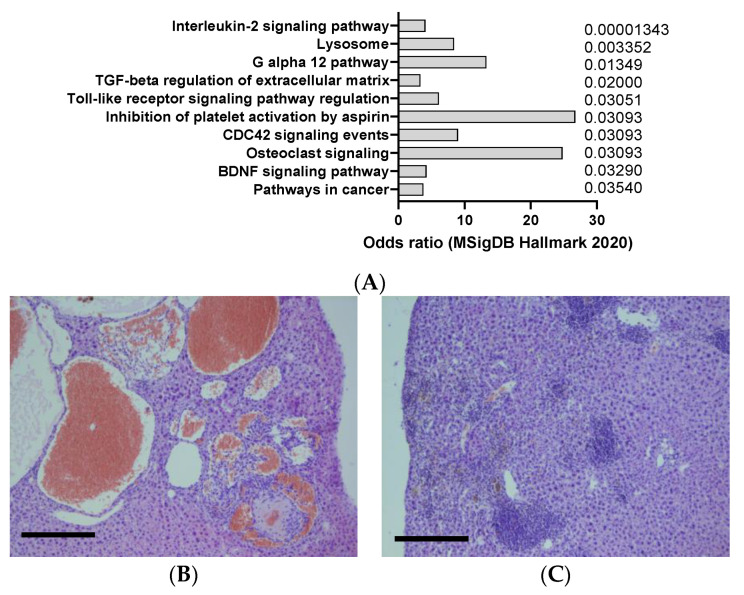
(**A**) mSigDB Hallmark 2020 pathway analysis of transcriptomics changes detected in urethane-induced lung cancer in mice, sorted by adjusted *p*-value (data adapted from GEO dataset nrGSE2514). (**B**) Example of liver cancer in urethane -induced animals, resembling hemangiosarcoma, according to hemalum–eosin staining (**C**) Example of liver inflammation in urethane -induced animals, according to hemalum–eosin staining. Scale bar: 100 µm (unpublished data).

Urethane is a mutagenic and carcinogenic compound that can induce lung adenocarcinoma in mice, especially in susceptible strains such as A/J and SWR/J. Urethane can cause point mutations, deletions, and chromosomal rearrangements in oncogenes and tumor suppressor genes, such as Kras, Egfr, and p53, that are frequently altered in human lung cancer [50]. Urethane is usually administered intraperitoneally or intravenously at doses ranging from 0.5 to 2 g/kg and can induce tumors within 6 to 12 months. Urethane-induced lung tumors are highly heterogeneous and can be classified into different histological subtypes, such as papillary, solid, mucinous, and mixed [51]. Urethane-induced lung tumors can also metastasize to other organs, such as the liver, kidney, and brain [52]. Urethane in mice appear to induce a clear interleukin 2 signaling response in the lung, but not other cytokines often dysregulated in lung cancer such as IL6, TNF-a, or interferon alpha (Figure 7A).

Urethane can also induce liver cancer (HCC) in adult and newborn mice exposed orally (by stomach tube), by intraperitoneal injection, by subcutaneous injection, and/or prenatally [53]. Urethane can also induce blood vessel tumors (hemangioma or hemangiosarcoma) of the liver, as observed in Figure 7B,C. The chemical can also cause cancer in the spleen, uterus, or unspecified sites in mice and hamsters exposed to urethane via the drinking water. Urethane is usually administered intraperitoneally or intravenously at doses ranging from 0.5 to 2 g/kg and can induce tumors within 6 to 12 months. Urethane-induced liver tumors are highly heterogeneous and can be classified into different histological subtypes, such as trabecular, pseudoglandular, and scirrhous [54].

However, the urethane-induced carcinogenesis also presents several limitations [55]. A study published in the *International Journal of Toxicology* suggests that the urethane carcinogenic cancer model may not be an appropriate model for studying the effects of urethane exposure on humans. The study states that the model does not accurately reflect the human disease and that the results obtained from this model may not be clinically relevant [56]. The study suggests that researchers should use alternative models that better mimic the human disease. 

## 3. Discussion

Preclinical cancer models are experimental systems that aim to mimic the biological and molecular features of human cancers and to test the efficacy and safety of potential anti-cancer therapies. They can be classified into two main categories: transplanted and spontaneous. Transplanted models involve the injection of tumor cells or tissues into immunocompetent or immunodeficient mice, whereas spontaneous models involve the induction of tumors in genetically engineered mice (GEMMs) or in mice treated with carcinogenic agents. Both types of models have advantages and limitations, depending on the research question and the tumor type. 

Transplanted models are easy to establish, manipulate, and reproduce and can be used to study the effects of different doses, routes, and schedules of treatment. However, transplanted models may not reflect the complexity and heterogeneity of human tumors. A major limitation of using preclinical models with transplanted tumors is the absence of interaction between the tumor and its surroundings—a feature that is present in natural models. Conversely, spontaneous genetically engineered models of cancer account for evolution and immune responses effectively but do not reproduce the history of disease in patients that is often induced by environmental stimuli or an infection. 

In comparison, spontaneous preclinical models induced by carcinogens enable more complex interactions between the tumor cells, surroundings, stromal and immune cells, hormones, cytokines, etc. Therefore, such models may offer a more comprehensive view of how a carcinogen, and ultimately cancer, impacts an animal model. Carcinogenic agents are chemicals or physical factors that can cause DNA damage, mutations, and epigenetic alterations in normal cells, leading to their malignant transformation. Some examples of carcinogenic agents were presented in this review in detail, showing that preclinical models induced by carcinogenic agents are widely used to study the mechanisms of carcinogenesis, tumor progression, and response to treatment [57]. 

There are many more environmental carcinogens that can increase the risk of cancer in humans. Some of the most common environmental carcinogens include tobacco smoke. Tobacco smoke contains more than 70 known carcinogens, including polycyclic aromatic hydrocarbons (PAHs), nitrosamines, and benzene. Exposure to tobacco smoke can cause lung cancer, bladder cancer, and many other types of cancer. Tobacco exposure was tested in mice using nicotine, which is the major addictive component of tobacco smoke. Although nicotine is generally thought to have limited ability to initiate cancer, it can induce cell proliferation and angiogenesis in a variety of systems, including in mice when nicotine is administered through intraperitoneal injections, or through over-the-counter transdermal patches [58].

Other carcinogenic agents include asbestos, which is a group of naturally occurring minerals that were once widely used in construction and manufacturing. Exposure to asbestos can cause lung cancer, mesothelioma, and other types of cancer in humans. Tests on several types of rodents, using different methods of exposure, have confirmed that asbestos causes cancer in animals. All forms of asbestos have caused tumors in animals, but the size and shape of the asbestos fibers influence the incidence of tumors. Smaller, straighter fibers seem more hazardous, perhaps because they are more likely to reach the deepest parts of the lungs [59]. Arsenic is also carcinogenic. Arsenic is a naturally occurring element that can be found in soil, water, and air. Exposure to arsenic in the drinking water can cause skin cancer, lung cancer, and other types of cancer. A study from the NTP laboratory that replicates how humans are exposed to arsenic through their whole lifetime found that mice exposed to low concentrations of arsenic in drinking water developed lung cancer [60]. Finally, pesticides are chemicals that are used to kill insects, rodents, and other pests that are also carcinogenic. Exposure to pesticides can cause cancer, including leukemia, lymphoma, and hemangiosarcoma in both humans and mice, as demonstrated for glyphosate [61].

## 4. Conclusions

It is important to emphasize that preclinical models induced by carcinogens can be challenging to create, control, and standardize and may have variable latency and penetrance, but it is worth pursuing as they resemble human responses closely. Chemically induced cancer models are an essential tool for studying the mechanisms and treatments of various types of cancers. However, the validity and reliability of these models depend on their similarity to human cancers in terms of molecular and cellular features [62]. In this review, the hallmarks of chemically induced preclinical cancer models are summarized, including genetic and epigenetic alterations, tumor microenvironment, angiogenesis, invasion and metastasis, and immune response [63]. The advantages and limitations of these models are also discussed, as well as the current challenges and future directions for their improvement. This review provides an overview of the state of the art of chemically induced cancer models and inspires new insights into cancer research. More work is still needed to provide a more comprehensive characterization of each model, as there were very few direct comparisons of omics characteristics of the carcinogen-induced models and corresponding human pathology.

To conclude, the choice of the preclinical model should be based on the specific aims and objectives of the study. Mimicking human exposure to carcinogenic agents through inhalation or drinking water in murine models appears to better recapitulate the regimen of carcinogen-induced cancers. However, further investigation, optimization, and validation in other models would be required to further standardize such approaches, in order to make them robust enough for drug testing and analysis of thorough molecular mechanisms.

## Figures and Tables

**Table 1:** List of commonly used carcinogenic agents in murine models.

Agent	Mechanism of Action	Organ Affected	Clinical Relevance
Azoxymethane (AOM)	Nitroso compound that requires metabolic activation to generate a DNA-reactive mutagen that induces colon cancer in mice and rats	Colon	A widely used carcinogen to study chemically induced colorectal carcinogenesis and is an agent for studying fulminant hepatic failure
Cerulein (also called ceruletide or caerulein) [19]	Ten amino acid oligopeptide that induces the secretion of pancreatic enzymes, causing inflammation and fibrosis	Pancreas	A potent carcinogen. It is used in paralytic ileus and as diagnostic aid in pancreatic malfunction. It is present in the skin of the Australian green tree frog
DEN (diethylnitrosamine) [20]	Nitrosamine that forms nitrosylated DNA adducts and activates oncogenes	Liver, colon, stomach, pancreas	A potent hepatocarcinogen and carcinogen for the digestive tract in rodents that is found in tobacco smoke
DMBA [21]	Polycyclic aromatic hydrocarbon (PAH) that can induce breast cancer by causing DNA damage	Breast, skin	A potent carcinogen for mice and humans, used to specifically quantify phlorotannins
DSS (dextran sulfate sodium) [22]	Sulfated polysaccharide that induces colitis-associated colorectal cancer by activating nuclear factor-kappa B and transforming growth factor-beta signaling pathways	Colon	A common environmental pollutant and dietary component that can cause inflammation and cancer in humans
Urethane [23]	Aromatic hydrocarbon that induces DNA damage and mutations in various tissues	Liver, lung, kidney, bladder, brain	A widely used solvent and industrial chemical that can cause cancer in humans

## Data Availability

Not applicable.
